# A possible role for miRNA silencing in disease phenotype variation in Swedish transthyretin V30M carriers

**DOI:** 10.1186/1471-2350-11-130

**Published:** 2010-09-14

**Authors:** Malin Olsson, Nina Norgren, Konen Obayashi, Violaine Plante-Bordeneuve, Ole B Suhr, Kristina Cederquist, Jenni Jonasson

**Affiliations:** 1 Department of Medicine, Umeå University, Umeå, Sweden; 2 Clinical Genetics, Umeå University Hospital, Umeå, Sweden; 3 Department of Diagnostic Medicine, Graduate School of Medical Sciences, Kumamoto University, Kumamoto, Japan; 4 Service de Neurologie, CHU Henri Mondor, Créteil, France

## Abstract

**Background:**

Familial amyloidosis with polyneuropathy (FAP) is an autosomal dominant disease caused by transthyretin (*TTR*) mutations, of which V30M (*TTR *c.148G > A, p.Val50Met, "Val30Met") is the most common. Swedish V30M carriers display later age at onset and lower penetrance compared to other populations.

**Methods:**

In the study, 130 Swedish V30M carriers (32 early, 30 late onset and 68 asymptomatic carriers) and 50 controls, 23 French symptomatic V30M carriers and 29 controls and 18 Japanese symptomatic V30M carriers and 29 controls were included. We aimed to identify additional genetic factors in the *TTR *gene and its surrounding region that could have an impact on phenotype.

**Results:**

We identified three SNPs (rs71383038, rs3794885 and rs62093482) with a significant difference in allele frequency between Swedish V30M carriers and controls. The two Swedish V30M homozygous patients present in the study also displayed homozygosity for the CA10 (rs71383038), A (rs3794885) and T (rs62093482) alleles in these SNPs. Hence, these alleles are present on the Swedish V30M haplotype. Of these, rs62093482 is located in the 3'UTR of *TTR *gene and thus more interesting since SNPs in the 3'UTR can affect gene expression levels by modifying microRNA (miRNA) targeting activity. miRNA target predictions revealed four potential miRNAs with predicted targets unique for the polymorphic allele.

**Conclusions:**

Our results are the first to show the presence of a 3'UTR polymorphism on the V30M haplotype in Swedish carriers, which can serve as a miRNA binding site potentially leading to down-regulated expression from the mutated *TTR *allele. This finding may be related to the low penetrance and high age at onset of the disease observed in the Swedish patient population.

## Background

Familial amyloidosis with polyneuropathy (FAP, OMIM +176300) is a fatal autosomal dominant disease caused by mutations in the *TTR *gene. *TTR *encodes transthyretin, a circulating and abundant tetramer that functions as a carrier protein of thyroid hormone and retinol binding protein. Transthyretin is mainly produced in the liver, but also in the brain and eye. Mutations in *TTR *cause the protein to dissociate into monomers that after misfolding assemble into amyloid fibrils deposits in a diversity of organs [1]. The main clinical symptom of FAP is progressive sensory-motor peripheral neuropathy, often associated with autonomic disturbances. Gastrointestinal dysfunction, renal failure and heart involvement with restrictive-hypertrophic cardiomyopathy and/or conduction disturbances are common complications. Genetically, FAP is a heterogeneous disease with approximately 100 amyloidogenic *TTR *missense mutations currently described. The most common *TTR *mutation V30M ("Val30Met", c.148G > A, p.Val50Met) is found sporadically worldwide, but also in endemic areas of Portugal, Japan and Sweden [2]. For V30M carriers there is considerable phenotypic variability both between and within populations and even within families. Maternal anticipation has been observed in families in the Swedish endemic area [3,4]. Northern Sweden has a high V30M carrier frequency (1.5% [5]) with an exceptionally low penetrance (5% before the age 40 [4]) and a high median age at onset, 56 years [5], leading to low incidence and prevalence. The French V30M population also exhibits low penetrance [6]. In contrast, endemic areas in Portugal have ten times lower carrier frequency than northern Sweden (0.18%, [7]), but the high penetrance (87% before the age 40 [8]) and early age at onset, 33 years [7], leads to high incidence and prevalence of the disease. Even though marked differences in phenotype are noted, haplotype analysis of the *TTR *gene region disclosed that Swedish V30M FAP patients all share a common founder [9]. A similar investigation of the French FAP population identified the presence of several founders for the *TTR *V30M mutation (Violaine Plante-Bordeneuve, CHU Henri Mondor, France, Personal communication). Portuguese and Japanese patients appear to share a common founder between the two populations [9,10]. No difference in disease penetrance has been noted between homozygous and heterozygous V30M carriers [11]. Unexpectedly, homozygous Swedish V30M carriers do not have a more aggressive disease than that of heterozygote V30M carriers.

The observed phenotypic variations among carriers of the same mutation indicate that additional, still unknown, genetic and epigenetic factors have an impact on phenotype and penetrance of this autosomal dominant trait. The two main aims of the present study were *i*) to identify additional genetic variants in the *TTR *gene and its surrounding region that might influence the phenotype of the disease with regards to age at onset and penetrance in northern Sweden, and *ii*) to compare these *TTR *gene variants between different populations. To address this, we compared allele frequencies for Swedish V30M FAP patients, asymptomatic V30M carriers and controls, and also Swedish with French and Japanese V30M FAP patients and controls.

## Methods

### Subjects and DNA samples

#### Swedish material

Sixty-two FAP *TTR *V30M patients referred for diagnostic testing at Clinical Genetics, University Hospital of Umeå, Sweden, between 1987 and 2007 were included in the study. Their FAP diagnose was based on presence of clinical symptoms consistent with the diagnosis, amyloid deposits in biopsy samples and the *TTR *V30M mutation. Patients were selected to represent two age at onset groups. In the early onset group (before age 40, n = 32), the mean age at onset was 32.0 ± 5.0 years, whereas the late-onset group (after age 50, n = 30) presented an average onset of 78.6 ± 6.1 years. Since 1986, all inhabitants in the County of Västerbotten in northern Sweden are invited to a health examination the year they turn 40, 50 and 60 years. Blood samples are collected and stored in The Northern Sweden Health and Disease Study (NSHDS) Cohort. From this cohort 3460 DNA samples were genotyped for the V30M allele, identifying 63 asymptomatic V30M carriers. All V30M carriers that since their contribution to the NSHDS cohort developed symptomatic FAP were excluded. Since all blood samples were obtained from individuals at the age of 60, and had been stored up to 10 years before they were analysed, we defined them as asymptomatic carriers. In addition, five asymptomatic V30M carriers present in the local FAP registry at the Department of Medicine were included, yielding in total 68 asymptomatic V30M carriers. The total number of *TTR *V30M carriers, both patients and asymptomatic carriers was 130. Fifty controls matched for age, geographical origin and gender were selected from the NSHDS cohort.

#### French material

DNA samples from 23 V30M FAP patients, with an age at onset between 29 and 74 years (55.6 ± 14.6), and 29 healthy controls were obtained from Department of Molecular Biology, CHU Bicêtre, Le Kremlin Bicêtre, France.

#### Japanese material

DNA samples from 18 V30M FAP patients, age at onset between 23 and 61 years (36.5 ± 12.1), and 29 healthy controls were obtained from the Department of Neurology, Kumamoto University Hospital, Kumamoto, Japan.

### Methods

DNA was extracted according to standard protocols. Genotyping of DNA samples from the NSHDS cohort was performed with mutation specific MGB probes, PCR and endpoint analysis (custom made TaqMan genotyping assay, probe and primer sequences available on request). In the Swedish material, all four *TTR *exons with a flanking intron/exon region of minimum 60 bp, regions 5'upstream (c.-136-1434 till c.-136-1, positions according to GenBank NM_000371.3) and 3'downstream (c.*349 till c.*349+79) including 14 single nucleotide polymorphisms (SNP), rs3764478, rs71383038, rs72922940, rs3764477, rs58616646, rs3794886, rs3794885, rs16962206, rs1551005, rs1800458, rs28933979 (V30M), rs1061978 and rs11541783, rs62093482 (SNPs according to NCBI and Ensembl), were analysed by sequencing (table 1). In addition, 19 known intronic SNPs, rs9304103, rs7231173, rs723744, rs1080093, rs1080094, rs17740912, rs58272364, rs13381331, rs1791225, rs3764476, rs10707844, ENSSNP11324634, rs7235277, rs3794884, rs1667250, rs1791226, rs1791227, rs1667251, rs59306196, with a minimum flanking region of 50 bp surrounding each SNP were also analysed by sequencing (table 1). In the French and Japanese samples only SNPs significant in the Swedish material were sequenced (table 1). PCR primer sequences and PCR conditions are available on request. Each primer was tailed with a universal M13 sequence and designed for universal PCR and sequencing conditions. PCR amplification was performed on a *GeneAmp PCR System 9700 *(Applied Biosystems) thermal cycler with AmpliTaq Gold (PE Applied Biosystems, USA) in 10 μl reactions containing 15-20ng of genomic DNA. Bidirectional sequencing was performed with dye terminator chemistry (Applied Biosystems) according to the manufacturer's instructions, scaled down to 1/16 in a 10 μl reaction. Sequencing reactions were electrophoresed on a 3730 × l DNA Analyzer using POP-7™ (Applied Biosystems), data analyzed with SeqScape Software v2.1 (ABI Prism, USA).

**Table 1:** SNPs in the TTR gene and the surrounding regions with polymorphisms present in the analysed Swedish samples.

			**Minor allele (frequency)**		
			
**dsSNP ID**	**Location NM_000371.3 (HGVS)**	**Location**	**Controls**	**V30M Carriers**	***P*-value^a^**
	
rs3764478	c.-136-1247G > T	5'upstream	T (0.09)	T (0.07)	
**rs71383038**	c.-136-1097_-1096delCA	5'upstream	CA9 (0.31)	CA9 (0.17)	0.0034
rs72922940	c.-136-1032A > G	5'upstream	G (0.07)	G (0.05)	
rs3764477	c.-136-1021G > A	5'upstream	A (0.00)	A (0.01)	
rs58616646	c.-136-1000C > T	5'upstream	T (0.00)	T (0.01)	
*	c.-136-697C > T	5'upstream	T (0.03)	T (0.01)	
**rs3794885**	c.-136-607A > T	5'upstream	T (0.32)	T (0.18)	0.0041
*	c.-136-546G > A	5'upstream	A (0.02)	A (0.03)	
rs723744	c.70-383G > T	intronic	T (0.31)	T (0.19)	
rs1800458	c.76G > A, p.G26R	exon 2	A (0.04)	A (0.02)	
rs28933979	c.148G > T, p.V50 M (V30M)	exon 2	G (0.00)	G (0.51)	
rs1080093	c.200+691C > G	intronic	G (0.31)	G (0.20)	
rs1080094	c.200+806A > G	intronic	G (0.30)	G (0.20)	
rs3764476	c.336+1242C > A	intronic	A (0.30)	A (0.20)	
ENSSNP11324634	c.336+1432A > G	intronic	G (0.00)	G (0.01)	
rs7235277	c.336+1655G > C	intronic	C (0.37)	C (0.18)	
rs3794884	c.337-1560T > G	intronic	G (0.31)	G (0.19)	
rs36204272	c.337-18G > C	intronic	C (0.00)	C (0.01)	
**rs62093482**	c.*261C > T	3'UTR	T (0.04)	C (0.48)	< 0.0001^b^

SNPs with significant differences in allele frequencies between V30M carriers and controls are shown in **bold**. Previously not reported polymorphisms are marked with a star (*). ^a^*P*-value ≤0.05 were considered statistical significant. *P*-value calculated by Fisher exact probability test. ^b^*P*-value significant after Bonferroni correction (*P*-value ≤0.003).

For sixteen SNPs only the wt allele were detected in the Swedish material, these are not included in the table: rs3794886, rs16962206, rs1551005, rs9304103, rs7231173, rs17740912, rs58272364, rs13381331, rs1791225, rs10707844, rs1667250, rs1791226, rs1791227, rs1667251, rs1061978, rs11541783.

### Statistics

To analyse differences in allele frequency between patient/carrier and control groups Fisher's exact probability test was performed using InStat v3.06 for Windows, GraphPad Software Inc. USA. All the *P*-values presented in the paper refer to two-sided T-test. *P*-values were also corrected for multiple testing using the Bonferroni method. For non corrected statistics a *P*-value ≤0.05 was considered significant, for corrected statistics a *P*-value ≤0.003.

### Ethics

The study was approved by the Ethical Committee of Umeå University, (Dnr 06-084M).

## Results

In order to analyse differences in allele frequencies that influence the phenotype of the disease with regards to age at onset and penetrance, we compared allele frequencies for all SNPs in Swedish FAP patients with early onset and late onset, all FAP patients, asymptomatic V30M carriers and all V30M carriers to controls. Significant differences in allele frequency were only detected when comparing all V30M carriers to controls. In the Swedish material two novel polymorphisms were found in the 5'upstream region (table 1). Neither of these showed any significant difference in allele frequency between V30M carriers and controls. Of the known SNPs analysed, a variant allele was detected in 17 (table 1). Of these, rs71383038, rs3794885 and rs62093482 showed a significant difference in allele frequency between V30M carriers and controls.

For SNP rs71383038 located upstream of the *TTR *gene start (c.-136-1097_-1096delCA) the Swedish V30M carriers showed a higher allele frequency of the major (CA10) allele compared to the controls (*P *= 0.0034, Table 1). The CA10 allele was present on at least one chromosome in all heterozygous V30M carriers. The two Swedish V30M homozygous patients present in the material also displayed homozygosity for CA10, confirming that the Swedish V30M haplotype also contains the CA10 allele. In the French patient material (a population with several V30M founders), the CA9 and CA10 alleles were evenly distributed and homozygosity for both alleles was found. This was significantly different from the French controls (*P *= 0.0015), where CA10 is more prevalent. None of the Japanese patients displayed homozygosity for CA9. However, no significant difference in the allele frequency of CA10 and CA9 was noted between patients and controls, probably due to limited sample size (n = 37).

For SNP rs3794885 located upstream of the *TTR *gene start (c.-136 -607A > T) A was identified as the major allele in the Swedish population. In addition, Swedish V30M carriers showed a higher allele frequency of the A allele compared to the controls (*P *= 0.0041, table 1). In analogy with the CA10 allele discussed above, the A allele is present on the V30M haplotype. However, for rs3794885 one patient heterozygous for V30M is homozygous for the T allele which is, likely explained either by a recombination event or *de novo *mutation on the original Swedish V30M haplotype. Also in the French population A is the major allele for rs3794885. In contrast to Swedish patients, French patients showed an overrepresentation of the T allele (*P *= 0.0058). The Japanese samples did not display any significant differences in the distribution of A and T alleles between patients and controls for rs3794885. This is probably due to both the low frequency of the T allele (4%) in this population and the small sample size.

For SNP rs62093482 located in the 3'UTR of the *TTR *gene (c.*261C > T) C is the major allele in the Swedish population (96%). However, Swedish V30M carriers showed a significantly higher allele frequency of the T allele compared to the controls (*P *< 0.0001, table 1). In the Swedish population rs62093482 is the only SNP that remains significant after correction for multiple testing. Also the T allele of rs62093482 can be assigned to the V30M haplotype. In the French population the allele frequencies of rs62093482 were in concordance with those of the Swedish population, yet the T allele was not detected among any of the French patients. In the Japanese population the T allele was not present at all.

### microRNA target predictions of polymorphism rs62093482

Previously published studies report that SNPs in the 3'UTR can affect gene expression levels by modifying miRNA targeting activity [12-15]. In this respect we found the 3'UTR polymorphism rs62093482 of particular interest. To investigate the presence of putative miRNA target sites that might be affected by this polymorphism, we analysed wild type (wt) and polymorphic sequences of the *TTR *3'UTR including the region c.*1-c.*349 using the miRNA target prediction programs MicroInspector 1.5 http://bioinfo.uni-plovdiv.bg/microinspector/[16] and the PITA algorithm by Segal Lab of Computational Biology http://genie.weizmann.ac.il/pubs/mir07/mir07_prediction.html[17]. We identified a total of five miRNAs with predicted target sites unique for the wt (C) allele and four miRNAs that were unique for the polymorphic T allele of rs62093482 (table 2). The miRNA hsa-miR-643, predicted to be unique for the polymorphic T allele, was the only miRNA to be identified using both MicroInspector and PITA. In an attempt to substantiate the predictions made in MicroInspector and PITA, we used RNAhybrid http://bibiserv.techfak.uni-bielefeld.de/rnahybrid/[18] and RegRNA http://regrna.mbc.nctu.edu.tw/[19] to cross analyse all nine identified miRNAs. To minimize false positive predictions we set up criteria that the predicted miRNA targets should fulfil. To be regarded as a potential miRNA target site, a predicted target site had to be unique for the wt or polymorphic alleles, respectively, in MicroInspector and/or PITA and also for this prediction to hold true with both programs used for the subsequent validation procedure. Based on these criteria all the identified miRNAs predicted to target wt 3'UTR (hsa-miR-593, hsa-miR-492, hsa-miR-1245, hsa-miR-581 and hsa-miR-142-3p) and one of the identified miRNAs predicted to target polymorphic 3'UTR (hsa-miR-384) were excluded (table 2). Of the remaining four miRNAs, all identified to be unique for the polymorphic 3'UTR, hsamiR-622 is the only miRNA still unique for the polymorphic target after the validation in both RNAhybrid and RegRNA. Regarding hsa-miR-643, hsa-miR-337-3p and hsa-miR-325, first identified as unique for the polymorphic allele, RNAhybrid predicted a target on both polymorphic and wt alleles (table 2). Based on thermodynamic properties of the miRNA:mRNA hybridisation analysed, the RNAhybrid predicted binding specificity to the wt allele is substantially weaker than that to the polymorphic allele, hence we still consider these miRNAs to be unique for the polymorphic allele.

**Table 2:** miRNA target predictions for rs62093482

**wt (C) allele**	**Identified with**	**Predicted binding to wt allele**	**Predicted binding to polymorphic allele**
		
hsa-miR-593	MicroInspector	RegRNA/RNAhybrid	RegRNA/RNAhybrid
hsa-miR-492	MicroInspector	RNAhybrid	RNAhybrid
hsa-miR-1245	PITA	RNAhybrid	RNAhybrid
hsa-miR-581	PITA	RegRNA/RNAhybrid	RNAhybrid^a^
hsa-miR-142-3p	PITA	RegRNA/RNAhybrid	RNAhybrid^a^
			
Polymorphic (T) allele	Identified with	Predicted binding to wt allele	Predicted binding to polymorphic allele
		
**hsa-miR-643**	MicroInspector/PITA	RNAhybrid^b^	RegRNA/RNAhybrid
**hsa-miR-337-3p**	MicroInspector	RNAhybrid^b^	RegRNA/RNAhybrid
hsa-miR-384	MicroInspector	RegRNA/RNAhybrid	RegRNA/RNAhybrid
**hsa-miR-622**	PITA		RegRNA/RNAhybrid
**hsa-miR-325**	PITA	RNAhybrid^b^	RegRNA/RNAhybrid

Underlined; predictions unique for wt or polymorphic target respectively in the subsequent validation procedure. In **bold**, miRNA target predictions that fulfil the criteria. ^a^Predicted for polymorphic target with only slightly weaker binding, C:G > U:G. ^b^Decreased binding specificity when predicted to wt target leading to reduced thermodynamic properties.

## Discussion

The previously published high V30M carrier frequency (8.3%) in the Lycksele subregion of the Västerbotten County in Sweden [5] could not be repeated in our study, which is based on a larger cohort. The actual carrier frequency of V30M in the Lycksele subregion was 2%. Our finding of 1.8% V30M carriers in our endemic area in Västerbotten is close to the V30M mutation frequency of 2.2% calculated from data reported from Västerbotten County by Holmgren *et al. *[5], and it is still 10 times higher than in Portugal.

The results for the SNP rs71383038 in the Swedish material showed that CA10 is significantly associated to V30M. This is similar to previous data from Portugal where CA10 is associated with V30M [20]. Neither this nor the other 5'upstream polymorphism (rs3794885) also significant associated to the V30M allele can be predicted to affect the binding of transcription factors according to TFSEARCH, Searching Transcription Factor Binding Sites (ver. 1.3) [21].

Our result show that the T allele of the 3'UTR polymorphism (rs62093482, positioned at c.*261) in all Swedish V30M carriers has the capacity to serve as a miRNA binding site. The miRNA target prediction tools available today have improved their accuracy since the first computational prediction program was developed in 2003. Yet, in order to increase the validity of our finding, we decided to use two different programs to screen for all known human miRNA against our chosen input target sequence (MicroInspector and PITA). Thereafter, we re-analysed the putative miRNAs, predicted by the above mentioned computational programs with two additional programs -RNAhybrid and RegRNA, to ensure that our putative miRNAs were unique for wt or polymorphic targets.

The miRNAs with predicted targets on the polymorphic allele that fulfilled our defined criteria were hsa-miR-643, hsa-miR-337-3p, hsa-miR-622 and hsa-miR-325. We consider hsa-miR-643 to be the best predicted candidate after re-evaluation of RNAhybrid's predictions based on the thermodynamic properties.

That the rs62093482 T allele has the capacity to serve as a miRNA binding site is especially intriguing since recent data show that motifs in the 3'UTR can be involved in gene regulation of certain genes via miRNA repression. Since all Swedish V30M carriers also carries this T allele we hypothesize that miRNA repression has an impact on the expression of the *TTR *V30M allele by partial silencing, which in turn could explain the low penetrance and high age at onset of disease observed in Swedish V30M carriers. Although the Swedish population as a group has a low penetrance, differences within the population still exists. The difference within the Swedish population might be explained by other factors such as differences in the regulation of the miRNA itself. Since miRNA functions as translational repressor and triggers mRNA degradation, one could expect plasma TTR levels to be lowered in Swedish V30M carriers due to the polymorphic 3'UTR. Data supporting this hypothesis was published earlier by Suhr *et al. *[22], showing significantly lower total plasma TTR levels in Swedish V30M carriers compared with Swedish controls, as measured by mass spectrometry analysis. In heterozygous TTR V30M carriers, variant TTR (V30M) protein contributed to only 42% of total TTR protein [22]. However, Westermark *et al. *were unable to find differences in total TTR concentration in plasma between Swedish V30M carriers and healthy controls in a study that included six V30M carriers [23]. These findings have been revised by Buxbaum *et al. *showing that there is a difference between Swedish TTR V30M carriers and controls, with higher TTR levels in controls than in V30M carriers [24]. These findings are consistent with studies from other populations that also have investigated the concentrations of TTR in plasma; i.e. patients with the V30M mutation have lower levels of TTR than controls. However, none of the latter studies have measured the relative amounts of mutated versus wild-type TTR in the same samples [24]. From these studies where only total plasma levels of TTR were measured, no conclusion about the variant TTR (V30M) levels can be drawn since TTR appears to have a self regulatory system of gene expression. This is supported by data from a previous study where intravenous administration of wt TTR to FAP patients resulted in suppression of the variant TTR (V30M) production [25]. If such an auto-regulatory loop exists, that keeps plasma TTR levels at a steady concentration, one can speculate that also partial transcriptional silencing of one allele (e.g. V30M due to the presence of allele specific miRNA binding site) could lead to upregulation of the other allele (wt) to maintain a stable total protein level. From the literature it is known that plasma TTR levels are age and sex dependent [26]. Therefore, patients and controls must be age and gender matched for comparison of TTR-concentration, and further studies are needed to elucidate if variant TTR concentration varies between V30M carriers and controls.

The effect of a miRNA on a target gene is dependent on the expression pattern and levels of the miRNA in different tissues. The expression patterns of the miRNAs identified in our study are to the best of our knowledge not described in the literature, and their respective expressions in liver and other target tissues in FAP are yet to be analysed. Future plans also include *in vitro *studies to evaluate the possibility of interaction between the polymorphic 3'UTR of the *TTR *gene and the predicted miRNAs (hsa-miR-643, hsa-miR-337-3p, hsa-miR-325 and hsa-miR-622) that fulfilled the criteria.

In this study we have focused on single-locus effects by allele frequencies of the three SNPs. Soares *et al. *has indicated that interactions among multiple-loci may explain the differences in phenotype [27]. Therefore, a multiple-loci analysis of our results together with adequate candidate genes would be of interest to evaluate the possibilities of gene-gene interactions relevant for disease development.

Epigenetic factors such as oxidative stress have been implicated in FAP as well as in other neurodegenerative diseases. Studies have shown that oxidative stress is increased in patients with amyloidosis [28,29], and in vitro studies have demonstrated that oxidative stress inhibits the initial rate and extent of amyloid fibril formation of wild-type as well as V30M transthyretin [30]. Our present data of a predicted novel miRNA targeting site adds to the already complex picture of FAP pathogenesis, and might contribute to the phenotypic diversity observed between V30M populations.

## Conclusion

Our analysis is the first to show that the T allele of the 3'UTR polymorphism rs62093482, present in all Swedish TTR V30M carriers, can serve as a miRNA binding site possibly leading to a down regulation of mutant TTR expression. This finding could be related to the low penetrance and high age at onset of the disease observed in the Swedish patient population.

## Competing interests

OBS has been a medical advisor to Alnylam Pharmaceuticals, Cambridge, Boston, MA, USA. The company has not been involved in or given any financial support to the present study.

## Authors' contributions

MO participated in planning and design of the study, acquisition of material and performed gene sequencing analysis and interpretation of the data and drafted and finalised the manuscript. NN contributed to the gene sequencing analysis, interpretation of data, contributed to the writing of the manuscript. KO contributed by acquisition of Japanese samples for the study. VP-B contributed by acquisition of the French samples for the study. OBS participated in planning and design of the study, acquisition of material, interpretation of the data and writing and finalisation of the manuscript. KC contributed to interpretation of the data, writing and finalisation of the manuscript. JJ contributed to interpretation of the data, writing and finalisation of the manuscript. All authors read and approved the final manuscript.

## Pre-publication history

The pre-publication history for this paper can be accessed here:

http://www.biomedcentral.com/1471-2350/11/130/prepub
